# T-DNA Analyzer: A Long-Read Sequencing Pipeline for Characterizing T-DNA Insertion Sites in Transgenic Crops

**DOI:** 10.3390/ijms27146201

**Published:** 2026-07-11

**Authors:** Yue Wan, Xiao-Ya Ma, Yi-Fan Yu, Zhan-Feng Si, Zhi-Cheng Shen, Yu-Xuan Ye

**Affiliations:** 1 State Key Laboratory of Rice Biology and Breeding, Key Laboratory of Biology of Crop Pathogens and Insects of Zhejiang Province, Institute of Insect Sciences, Zhejiang University, Hangzhou 310058, China; 2 The Rural Development Academy, Zhejiang University, Hangzhou 310058, China; 3 Zhejiang University Zhongyuan Institute, Zhengzhou 450000, China

**Keywords:** T-DNA insertion, long-read sequencing, transgenic crop, Oxford Nanopore, PacBio, GMO characterization, structural variation, gene impact annotation, bioinformatics pipeline

## Abstract

Molecular characterization of the transferred DNA (T-DNA) insertion sites is required for the safety assessment of genetically modified (GM) crops, yet conventional PCR-based methods are labor-intensive and limited in their ability to resolve complex structural variations. We present T-DNA Analyzer, an integrated bioinformatics pipeline that transforms long-read sequencing data (PacBio HiFi or Oxford Nanopore) into a comprehensive insertion site report. The pipeline implements a host-derived read filter that subtracts host-homologous vector regions to eliminate false-positive chimeric read calls; a multi-segment fusion detection algorithm that resolves complex T-DNA integration architectures; and a deletion gap gene impact analysis that identifies genes affected by host genome deletions at the integration site. Validation on maize and cotton datasets demonstrated that the host-derived filter excluded 86.4% of false-positive reads while retaining all true chimeric reads, and the fusion detection algorithm successfully reconstructed a two-copy tandem T-DNA repeat within a single long read. T-DNA Analyzer provides automated, reproducible molecular characterization designed to support regulatory molecular characterization and is freely available as open-source software.

## 1. Introduction

Regulatory agencies worldwide, including the European Food Safety Authority (EFSA), the U.S. Food and Drug Administration (FDA), and their counterparts in China, Brazil, and India, require comprehensive molecular characterization of each genetically modified (GM) crop transformation event before commercial release [1]. This characterization must determine the transferred DNA (T-DNA) insertion sites, copy number, transgene structural integrity, flanking host sequences, and the potential genotoxic impact on endogenous genes [2]. With GM crops cultivated across 190.4 million hectares in 26 countries and delivering substantial reductions in pesticide use [3], the demand for reliable, scalable characterization methods continues to grow.

*Agrobacterium tumefaciens*-mediated transformation remains the most widely used method for generating transgenic plants. The T-DNA is delivered into the plant nucleus as a single-stranded molecule and integrates into the host genome through illegitimate recombination mediated by the host DNA repair machinery [4,5]. The integration process is inherently imprecise. T-DNA inserts can be truncated, rearranged, tandemly repeated, or intermingled with filler DNA of host or vector origin [6,7]. T-DNA integration is frequently accompanied by host genome deletions ranging from a few base pairs to several kilobases, as well as more complex chromosomal rearrangements including inversions and translocations [8,9]. These complexities pose significant analytical challenges for traditional molecular characterization methods.

Conventional approaches to T-DNA insertion site characterization, including Southern blotting, thermal asymmetric interlaced PCR (TAIL-PCR), inverse PCR, and genome walking, have well-documented limitations [10,11]. These methods are labor-intensive, require event-specific primer design, are prone to PCR amplification biases, and frequently fail to resolve complex integration patterns involving multiple T-DNA copies, vector backbone sequences, or large structural variations [12]. Next-generation sequencing (NGS) has enabled genome-wide characterization of transgenic events through paired-end whole-genome sequencing and targeted capture sequencing [13,14]. However, short-read NGS technologies (approximately 150 to 300 bp) are fundamentally limited in their ability to span repetitive elements, resolve tandem duplications, and phase complex structural variations that span multiple kilobases [15].

Third-generation long-read sequencing technologies, specifically Pacific Biosciences (PacBio) single-molecule real-time (SMRT) sequencing and Oxford Nanopore Technologies (ONT) sequencing, have emerged as tools for resolving complex genomic architectures [16,17]. PacBio HiFi reads offer high accuracy (at least 99.9%) with read lengths of 10 to 25 kb, while ONT reads can exceed 100 kb, providing the contiguity needed to span entire T-DNA insertions along with substantial flanking host sequences in a single read. Recent studies have demonstrated the utility of long-read sequencing for characterizing T-DNA insertions in maize [18,19], Arabidopsis [9], and other crop species [20].

However, these studies relied on customized, ad hoc bioinformatics workflows that are not readily transferable across laboratories or species. None have provided an integrated solution that combines host-derived read filtering, multi-fragment fusion detection, deletion gap gene impact analysis, and publication-quality visualization within a single automated pipeline.

**Figure 1:**
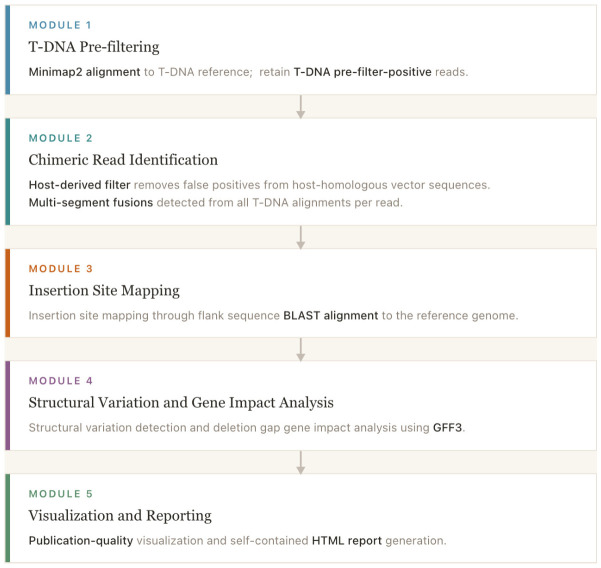
Schematic overview of the T-DNA Analyzer pipeline.

To address these gaps, we developed T-DNA Analyzer, an open-source bioinformatics pipeline for characterizing T-DNA insertion events from long-read sequencing data (Figure 1). The pipeline integrates a host-derived read filter to eliminate false-positive calls from host-homologous vector sequences, a multi-segment fusion detection algorithm to resolve complex integration patterns, and a deletion gap gene impact analysis to identify genes affected by host genome deletions at the integration site. It produces publication-quality, colorblind-safe figures and a self-contained HTML report suitable for regulatory documentation.

In this paper, we describe the design and implementation of T-DNA Analyzer. We demonstrate its performance on maize and cotton data and discuss its potential for standardizing transgenic crop molecular characterization.

## 2. Results

### 2.1. Maize HiFi Case: Validation of Host-Derived Read Filter

A long-read sequencing dataset was generated from a maize (*Zea mays*) transgenic event using PacBio HiFi sequencing on the Revio platform. The sequencing produced 2,022,140 raw reads, which were analyzed using the Zm-A188-REFERENCE-KSU-1.0 reference genome [21] and its accompanying GFF3 gene annotation.

The minimap2 pre-filter identified 59 reads with at least 500 bp T-DNA alignment from the 2,022,140 input reads. The host-derived read filter detected T-DNA regions with homology to the maize genome (≥90% identity and ≥300 bp alignment to the MaizeGDB A188 reference) and classified 51 of the 59 pre-filter-positive reads as host-derived, corresponding to a false-positive exclusion rate of 86.4%. The remaining 8 reads were processed through chimeric read detection, yielding 8 chimeric reads all mapping to the same genomic locus on chromosome 10 (Table 1 and Table 2). The pipeline is highly efficient; processing this dataset required approximately 5 min using 8 CPU threads and 32 GB of RAM.

Of the eight confirmed chimeric reads, six possessed both LB and RB flanking sequences (chimera type 1: both flanks), one possessed only an RB flank (chimera type 2: RB-only), and one possessed only an LB flank (chimera type 3: LB-only). The RB positions clustered at 108,008,084 bp and LB positions clustered at 108,008,360 bp, with a 276 bp gap between junction coordinates indicating a host genome deletion at the integration site [4,6]. T-DNA coverage among the supporting reads ranged from 45.9% to 98.5% of the full-length reference. The representative read (m84179_260228_110539_s4/104338713/ccs) contained 10,933 bp (98.5%) of the T-DNA sequence. Reads were observed in both forward and reverse orientations (2 forward, 6 reverse). Gene impact analysis of the 276 bp deletion gap classified the site as intergenic. All eight supporting reads were clustered into a single insertion site (Site 1) within the 15,000 bp clustering window, located on chromosome 10 at position 108,008,084 to 108,008,360.

### 2.2. Cotton Resolved Insertion Case: Validation of Multi-Segment Fusion Detection

To demonstrate the pipeline’s ability to resolve complex insertion architectures, we re-analyzed a previously characterized transgenic cotton (*Gossypium hirsutum*) event using T-DNA Analyzer. The transgenic event AHS10 contains two copies of the T-DNA inserted at a single genomic locus on chromosome A05 [22]. The T-DNA construct (12,879 bp) includes the *cry1Ab/vip3Aa* insect resistance fusion gene, the *cp4-epsps* glyphosate tolerance gene, and the *bar* glufosinate tolerance selectable marker. A single 27,034 bp chimeric read spanning the entire insertion with both LB and RB flanking host sequences was provided as input, along with the TM-1 reference genome [23]. This multi-copy event structure matches experimental validation previously reported by Mou et al. [22].

**Figure 2:**
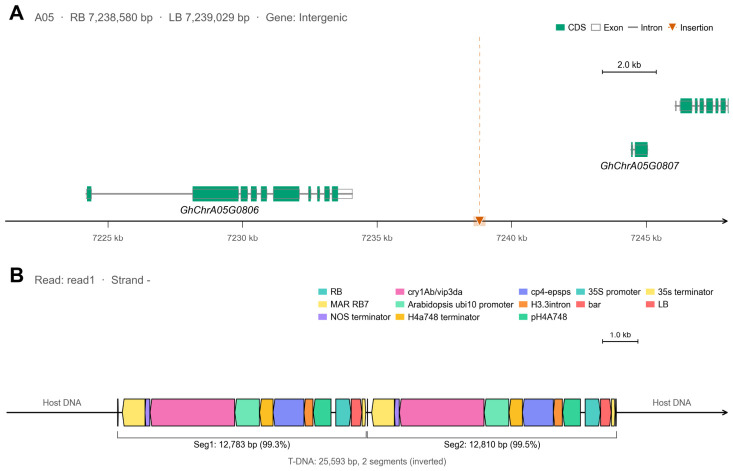
T-DNA Analyzer output figure for the cotton two-segment fusion insertion site: (**A**) Genomic context panel showing the insertion site on cotton chromosome A05 with flanking gene models. CDS regions are shown as green filled boxes, exons as white outlined boxes, introns as grey connecting lines, and the insertion site as a dashed vermillion line with a triangular marker. (**B**) T-DNA structure panel depicting the two T-DNA segments, with annotated element arrows, host DNA flanking regions, and per-segment boundary lines.

The LB junction was mapped to position 7,239,029 and the RB junction to position 7,238,580 on chromosome A05, with a 449 bp gap between junction coordinates indicating a host genome deletion at the integration site. Gene impact analysis of the 449 bp deletion gap classified the site as intergenic, with no functional genes deleted (Figure 2A).

The pipeline detected a two-segment T-DNA fusion event, consistent with the two-copy insertion at this locus. Both segments exhibited near-full-length coverage of the T-DNA reference: Segment 1 covered 12,783 bp (99.3%) from reference position 27 to 12,809, and Segment 2 covered 12,810 bp (99.5%) from reference position 49 to 12,858 (Figure 2B; Table 3). The two T-DNA segments were arranged consecutively along the read with overlapping reference coordinates, forming a tandem T-DNA repeat.

## 3. Discussion

T-DNA Analyzer addresses a critical bottleneck in the molecular characterization workflow for GM crops by providing an integrated, automated pipeline that transforms raw long-read sequencing data into a comprehensive insertion site report. The pipeline’s design emphasizes automation, accuracy, interpretability, and portability.

A key challenge in T-DNA insertion site detection is that plant transformation vectors frequently incorporate endogenous promoters, introns, and other regulatory elements from the target species [24]. When a transgenic plant is sequenced, host genomic reads containing these elements can cross-align to the T-DNA vector and be misidentified as chimeric reads, leading to false-positive insertion site calls [12]. The host-derived read filter addresses this by detecting host-homologous T-DNA intervals and subtracting them from each read’s alignment profile. Reads whose remaining non-host T-DNA contigs are below 500 bp are classified as host-derived and excluded. This criterion is grounded in biology: genuine chimeric reads carry a continuous segment of unique vector sequence spanning from one border to the other, consisting predominantly of exogenous sequences absent from the host genome. Host genomic reads, in contrast, produce only small, scattered fragments at the edges of host-homologous regions. In the maize dataset, this filter excluded 51 of 59 pre-filter-positive reads (86.4%) while retaining all 8 true chimeric reads, demonstrating that the two populations are cleanly resolved without manual curation.

The use of long-read sequencing enables the detection of complex T-DNA integration architectures that are inaccessible to short-read approaches [15]. Short reads cannot resolve tandem T-DNA repeats because the repetitive sequences produce reads that map equally well to multiple positions. A long read spanning the entire rearrangement provides unambiguous phase information. However, most alignment-based pipelines retain only the single best alignment per read, which discards evidence of secondary T-DNA copies. T-DNA Analyzer’s fusion detection algorithm instead processes all T-DNA alignments and assembles them into coherent multi-segment structures. Without this, secondary T-DNA copies within the same read are invisible, and a complex multi-copy insertion event would be mischaracterized as a simple single-copy insertion, missing information critical for safety assessment. In the cotton AHS10 event, the pipeline identified two near-full-length T-DNA segments (each >99% coverage) arranged as a tandem repeat within a single 27 kb read, consistent with the previously characterized two-copy insertion at this locus [22]. The algorithm additionally resolves overlapping coordinates between adjacent segments and applies a BLAST refinement step to improve junction precision to ±1–10 bp [25]. Tandem T-DNA repeats have been documented in multiple transgenic events [6,7,26] and are thought to arise from template switching during DNA repair [4].

Conventional approaches typically report a single gene impact classification for the entire insertion event. However, because T-DNA insertions are frequently accompanied by host genome deletions, the gap between junction coordinates may span entire genes [8,9]. T-DNA Analyzer analyzes the full deletion gap for gene content: genes completely contained within the gap are reported as “Gene Deletion,” while partially overlapped genes are classified by the most severely affected feature (CDS > exon > intron > intergenic). This provides a more complete genotoxicity assessment, particularly relevant for regulatory submissions [27].

The pipeline has been validated on maize (monocot, ~2.3 Gb genome, A188 assembly [21]) and cotton (dicot, ~2.3 Gb allotetraploid genome, TM-1 assembly [23]), demonstrating applicability across major crop lineages. All algorithmic parameters are configurable via a YAML file, allowing users to adjust thresholds without modifying source code. Whole-genome long-read sequencing eliminates the probe design, hybridization, and amplification steps required by enrichment-based methods such as LIFE-Seq [20], providing direct alignment evidence with quantifiable, reproducible metrics. As long-read sequencing becomes increasingly accessible and cost-effective [28], tools such as T-DNA Analyzer have the potential to standardize the molecular characterization workflow, reducing the time from transformation event generation to regulatory submission.

Several limitations warrant discussion. The pipeline requires a pre-assembled reference genome, which may not be available for orphan crops. The insertion site clustering algorithm uses a fixed distance threshold (15,000 bp by default, configurable), which may be suboptimal for complex events with clustered multi-site insertions. T-DNA Analyzer does not perform de novo assembly of the integrated transgene locus; integration with tools such as Canu [29] or Flye [30] could provide additional structural resolution. Raw ONT reads with 5–10% error rates may reduce alignment sensitivity for short flank sequences [28]; we recommend using PacBio HiFi reads or error-corrected ONT reads. Furthermore, highly repetitive host loci (e.g., centromeres, telomeres) or extremely short, truncated T-DNA insertions may challenge flank mapping and complex structural resolution.

## 4. Materials and Methods

### 4.1. Pipeline Architecture and Implementation

T-DNA Analyzer is implemented in Python (version 3.8 or later) and orchestrates two external sequence alignment tools: minimap2 (version 2.24 or later) for structural long-read alignment [31] and BLAST+ (version 2.15 or later) for terminal coordinate refinement and short-sequence alignment [25]. Sequence input/output and manipulation are handled by Biopython (version 1.81 or later) [32], while data analysis and tabulation rely on pandas (version 1.5 or later) and NumPy (version 2.0 or later). Figures are generated using matplotlib (version 3.5 or later) with configurable output formats. The pipeline is designed for cross-platform compatibility (Windows, macOS, and Linux) and supports multi-threaded parallel execution.

The pipeline consists of five computational modules executed sequentially:

- Module 1: T-DNA Pre-filtering. Raw long reads are aligned to the T-DNA reference sequence using minimap2. The pipeline supports both PacBio HiFi (map-hifi preset) and Oxford Nanopore (map-ont preset) read types, with automatic platform detection from read length distributions or manual specification via the configuration file. Reads with fewer than 500 bp of cumulative T-DNA alignment (default, adjustable via config.default.yaml) are discarded, removing non-transgenic reads while preserving partial T-DNA-containing reads.
- Module 2: Chimeric Read Identification. Host-homologous T-DNA regions are detected by BLASTN alignment of the T-DNA sequence against the host genome. Host-derived reads are filtered by contig-based subtraction of host-homologous intervals (Section 4.2). Filtered reads are aligned to the T-DNA reference using BLASTN (e-value ≤ 1 × 10^−100^) to identify chimeric reads, which are classified into three types (both flanks, Left Border (LB)-only, Right Border (RB)-only) and screened for multi-segment T-DNA fusions (Section 4.3). A subsequent BLASTN refinement step using high-stringency parameters (reward = 1, penalty = −5) adjusts terminal coordinates to ±1–10 bp precision.
- Module 3: Insertion Site Mapping. Flanking host sequences (500 bp from each T-DNA junction) are extracted from chimeric reads and aligned to the reference genome using BLASTN with short-sequence-optimized parameters (word_size = 11, reward = 1, penalty = −3). A genomic blacklist derived from host-like T-DNA region detection prevents spurious alignments. A full-length flank fallback mechanism handles cases with large filler DNA. Unknown filler DNA sequences of unmapped origins are simply retained and reported as unaligned sequence gaps between the host flank and the T-DNA segment within the chimeric read.
- Module 4: Structural Variation and Gene Impact Analysis. T-DNA structural variations are classified by comparing aligned T-DNA coverage to the full-length reference. The deletion gap between LB and RB junction coordinates is analyzed against GFF3 gene models to identify genes fully deleted or partially affected, with impacts classified as Gene Deletion, CDS, exon, intron, or intergenic (Section 4.4).
- Module 5: Visualization and Reporting. Publication-quality, colorblind-safe multi-panel figures using the Okabe-Ito palette and a self-contained HTML report with interactive sequence visualizations are generated for each insertion site (Section 4.5).

### 4.2. Host-Derived Read Filter Algorithm

The host-derived read filter addresses a critical source of false positives in T-DNA insertion site detection. When the T-DNA vector contains sequences with homology to the host genome, host genomic reads can cross-align to these regions. Such sequences include endogenous promoters (for example, ubiquitin promoters), introns, or vector backbone fragments derived from the host species. The cross-aligned reads may be erroneously classified as chimeric reads bearing T-DNA insertions.

The filter operates through three computational steps:

- Detection. The full T-DNA sequence is aligned against the reference genome using BLASTN (minimum identity at least 90%, minimum alignment length at least 300 bp). T-DNA intervals with significant genomic homology are recorded, and nearby intervals (less than 5 kb apart) that map to the same chromosome are merged.
- Subtraction. For each read that passed the initial minimap2 pre-filter, all T-DNA reference intervals aligned by the read are collected and merged. The host-homologous T-DNA intervals (from step 1) are then subtracted from these merged intervals, yielding a set of non-host T-DNA contigs.
- Classification. A read is classified as host-derived if its largest non-host T-DNA contig is shorter than 500 bp. This contig-based criterion is stringent. Genuine chimeric reads carry a large continuous block of unique T-DNA sequence spanning at least several hundred base pairs. Host DNA reads produce only small, scattered alignment fragments (typically less than 250 bp) derived from short border repeats or alignment noise at the edges of host-homologous regions.

### 4.3. Multi-Segment T-DNA Fusion Detection

Fusion detection is integrated into the chimeric read identification module. For each read, all BLAST alignments to the T-DNA reference exceeding 100 bp are retained rather than only the single best alignment. These alignment segments are sorted by their read-level coordinates. Segments are filtered to remove those covering less than 10% of the full T-DNA reference length (default, configurable), which are considered alignment noise rather than genuine additional T-DNA copies.

When two or more non-overlapping T-DNA segments are present in the same read, a fusion event is recorded. Each segment is annotated with its T-DNA reference coordinates, strand orientation, and coverage classification (full-length, 5-prime partial, 3-prime partial, or internal). Adjacent segments with overlapping read coordinates are resolved by assigning the shared base to the earlier segment and shifting the later segment one position downstream. Strand orientation is determined from the minimap2 PAF alignment and verified against the T-DNA reference sequence. A subsequent BLASTN refinement step (reward = 1, penalty = −5) adjusts terminal coordinates of each segment to ±1–10 bp precision.

### 4.4. Deletion Gap Gene Impact Analysis

Gene impact annotation focuses on the deletion gap between the LB and RB junction coordinates on the host chromosome. T-DNA integration is frequently accompanied by host genome deletions ranging from tens of base pairs to several kilobases [4,6]. When both junctions are mapped, the pipeline queries the GFF3 gene model to identify genes affected by the deletion. Two categories are reported: genes whose entire span falls within the gap (complete gene deletion, classified as “Gene Deletion”), and genes partially overlapped by the gap. For partially affected genes, the overlap is classified by feature severity—CDS overlaps (most severe), followed by non-coding exon overlaps, intronic overlaps, and intergenic regions. This hierarchy reflects the regulatory significance of gene disruption in GM crop safety assessments [27].

### 4.5. Visualization and Reporting

For each insertion site, T-DNA Analyzer generates a publication-quality figure showing the genomic context (chromosome coordinates, flanking gene models, and the insertion site) and the T-DNA structure within the chimeric read (annotated elements, host flanking regions, and filler DNA). For multi-segment T-DNA fusions, per-segment boundaries and coverage are additionally displayed. All figures use the Okabe-Ito colorblind-safe palette [33] and are exportable in multiple formats (PDF, PNG, SVG, TIFF). A self-contained HTML report is generated with figures, interactive sequence visualizations, structured conclusions, and comprehensive data tables suitable for regulatory documentation.

The pipeline parameters used in this study are the default values provided in config.default.yaml. All thresholds—including alignment minima, BLAST e-values, clustering window size, and host-homology detection criteria—can be adjusted via this file without modifying source code.

## 5. Conclusions

We have developed T-DNA Analyzer, an integrated bioinformatics pipeline for comprehensive characterization of T-DNA insertion sites in transgenic crops using long-read sequencing data. The pipeline implements five computational modules that together transform raw sequence reads into a publication-quality analysis report with a single command-line invocation.

The key methodological contributions are a host-derived read filter that eliminates false-positive calls from host-homologous vector sequences, a multi-segment T-DNA fusion detection algorithm that resolves complex integration architectures using long-read contiguity, and a deletion gap gene impact analysis that identifies genes affected by the host genome deletion at the integration site.

Validation on both maize PacBio HiFi and cotton ONT datasets demonstrated that T-DNA Analyzer accurately characterizes both simple full-length insertions and complex multi-segment T-DNA fusions. Its contig-based filter effectively excludes host-derived false-positive reads. The pipeline generates comprehensive reports across monocot and dicot crop species.

As long-read sequencing becomes increasingly accessible, fully automated pipelines such as T-DNA Analyzer can standardize the molecular characterization workflow for transgenic crops, reducing the time from transformation event generation to regulatory submission while improving the comprehensiveness and reproducibility of safety assessments.

## Figures and Tables

**Table 1:** Host-derived read filter performance on the maize HiFi dataset.

Metric	Value
Total input reads	2,022,140
Pre-filter-positive reads	59
Host-derived reads excluded	51 (86.4% of pre-filter positives)
Chimeric reads confirmed	8 (100% true-positive retention)
Insertion sites identified	1

**Table 2:** Summary of chimeric reads identified after host-derived filtering in the maize HiFi dataset using the MaizeGDB A188 reference genome.

Read ID	Chromosome	LB Position	RB Position	T-DNA Coverage	Chimera Type
read_104338713	chr10	108,008,360	108,008,084	98.50%	Both flanks
read_155649939	chr10	108,008,360	—	91.40%	LB only
read_57095473	chr10	108,008,360	108,008,084	98.50%	Both flanks
read_165415752	chr10	108,008,360	108,008,084	98.50%	Both flanks
read_179377898	chr10	—	108,008,084	98.50%	RB only
read_02380264	chr10	108,008,360	108,008,084	98.50%	Both flanks
read_31855008	chr10	108,008,360	—	45.90%	LB only
read_64360966	chr10	108,008,360	108,008,084	98.50%	Both flanks

**Table 3:** Summary of the cotton resolved insertion event analysis results.

Metric	Value
Read length	27,034 bp
T-DNA construct length	12,879 bp
Fusion segments detected	2
Segment 1 coverage	12,783 bp (99.3%; positions 27–12,809)
Segment 2 coverage	12,810 bp (99.5%; positions 49–12,858)
Chromosome	A05
LB junction	7,239,029
RB junction	7,238,580
Deletion gap	449 bp
Gene impact	Intergenic

## Data Availability

The data presented in this study are openly available in GitHub at https://github.com/yyxx0037/T-DNA-Analyzer (accessed on 8 July 2026).

## Abbreviations

The following abbreviations are used in this manuscript:

GM	Genetically Modified
T-DNA	Transfer DNA
SV	Structural Variation
LB	Left Border
RB	Right Border

## References

[1] EFSA Panel on Genetically Modified Organisms (GMO) (2010). Guidance on the environmental risk assessment of genetically modified plants. EFSA J..

[2] Kovalic D., Garnaat C., Guo L., Yan Y., Groat J., Silvanovich A., Ralston L., Huang M., Tian Q., Christian A. (2012). The use of next generation sequencing and junction sequence analysis bioinformatics to achieve molecular characterization of crops improved through modern biotechnology. Plant Genome.

[3] Brookes G., Barfoot P. (2020). Environmental impacts of genetically modified (GM) crop use 1996–2018: Impacts on pesticide use and carbon emissions. GM Crops Food.

[4] Gelvin S.B. (2021). Plant DNA repair and Agrobacterium T−DNA integration. Int. J. Mol. Sci..

[5] Tzfira T., Li J., Lacroix B., Citovsky V. (2004). Agrobacterium T-DNA integration: Molecules and models. Trends Genet..

[6] Zhu Q.-H., Ramm K., Eamens A.L., Dennis E.S., Upadhyaya N.M. (2006). Transgene structures suggest that multiple mechanisms are involved in T-DNA integration in plants. Plant Sci..

[7] De Buck S., Jacobs A., Van Montagu M., Depicker A. (1999). The DNA sequences of T-DNA junctions suggest that complex T-DNA loci are formed by a recombination process resembling T-DNA integration. Plant J..

[8] Forsbach A., Schubert D., Lechtenberg B., Gils M., Schmidt R. (2003). A comprehensive characterization of single-copy T-DNA insertions in the *Arabidopsis thaliana* genome. Plant Mol. Biol..

[9] Pucker B., Kleinbölting N., Weisshaar B. (2021). Large scale genomic rearrangements in selected *Arabidopsis thaliana* T-DNA lines are caused by T-DNA insertion mutagenesis. BMC Genom..

[10] Liu Y.-G., Chen Y. (2007). High-efficiency thermal asymmetric interlaced PCR for amplification of unknown flanking sequences. Biotechniques.

[11] Ochman H., Gerber A.S., Hartl D.L. (1988). Genetic applications of an inverse polymerase chain reaction. Genetics.

[12] Guo B., Guo Y., Hong H., Qiu L.-J. (2016). Identification of genomic insertion and flanking sequence of G2-EPSPS and GAT transgenes in soybean using whole genome sequencing method. Front. Plant Sci..

[13] Wang F., Lu S., Xu W., Yang L. (2025). Deciphering the complex molecular architecture of the genetically modified soybean FG72 through paired-end whole genome sequencing. Food Chem. Mol. Sci..

[14] Magembe E.M., Li H., Taheri A., Zhou S., Ghislain M. (2023). Identification of T-DNA structure and insertion site in transgenic crops using targeted capture sequencing. Front. Plant Sci..

[15] Sedlazeck F.J., Rescheneder P., Smolka M., Fang H., Nattestad M., Von Haeseler A., Schatz M.C. (2018). Accurate detection of complex structural variations using single-molecule sequencing. Nat. Methods.

[16] Wenger A.M., Peluso P., Rowell W.J., Chang P.-C., Hall R.J., Concepcion G.T., Ebler J., Fungtammasan A., Kolesnikov A., Olson N.D. (2019). Accurate circular consensus long-read sequencing improves variant detection and assembly of a human genome. Nat. Biotechnol..

[17] Jain M., Olsen H.E., Paten B., Akeson M. (2016). The Oxford Nanopore MinION: Delivery of nanopore sequencing to the genomics community. Genome Biol..

[18] Liu Q., Wang Q., Ning L., Chen Z., Zhang C., Liu Y., Qian B., Guo J., Yin Y. (2024). Efficient identification of genomic insertions and surrounding regions in two transgenic maize events using third-generation single-molecule nanopore sequencing technology. Sci. Rep..

[19] Boutigny A.-L., Fioriti F., Rolland M. (2020). Targeted MinION sequencing of transgenes. Sci. Rep..

[20] Zhang H., Li R., Guo Y., Zhang Y., Zhang D., Yang L. (2022). LIFE-Seq: A universal L arge I ntegrated DNA F ragment E nrichment Seq uencing strategy for deciphering the transgene integration of genetically modified organisms. Plant Biotechnol. J..

[21] Lin G., He C., Zheng J., Koo D.-H., Le H., Zheng H., Tamang T.M., Lin J., Liu Y., Zhao M. (2021). Chromosome-level genome assembly of a regenerable maize inbred line A188. Genome Biol..

[22] Mou Q., Zhang J., Si Z., Jin S., Zhang W., Zhang T. (2026). Transgenic Lepidopteran-Pests-Resistant and Herbicide-Tolerant Cotton Through Transfer of *Cry1Ab-vip3Aa* and *Cp4-epsps+ bar* Genes. Plant Biotechnol. J..

[23] Yan H., Han J., Jin S., Han Z., Si Z., Yan S., Xuan L., Yu G., Guan X., Fang L. (2025). Post-polyploidization centromere evolution in cotton. Nat. Genet..

[24] Podevin N., Du Jardin P. (2012). Possible consequences of the overlap between the CaMV 35S promoter regions in plant transformation vectors used and the viral gene VI in transgenic plants. GM Crops Food.

[25] Altschul S.F., Gish W., Miller W., Myers E.W., Lipman D.J. (1990). Basic local alignment search tool. J. Mol. Biol..

[26] Windels P., De Buck S., Van Bockstaele E., De Loose M., Depicker A. (2003). T-DNA integration in Arabidopsis chromosomes. Presence and origin of filler DNA sequences. Plant Physiol..

[27] Schnell J., Steele M., Bean J., Neuspiel M., Girard C., Dormann N., Pearson C., Savoie A., Bourbonniere L., Macdonald P. (2015). A comparative analysis of insertional effects in genetically engineered plants: Considerations for pre-market assessments. Transgenic Res..

[28] Yildiz G., Zanini S.F., Afsharyan N.P., Obermeier C., Snowdon R.J., Golicz A.A. (2023). Benchmarking Oxford Nanopore read alignment-based insertion and deletion detection in crop plant genomes. Plant Genome.

[29] Koren S., Walenz B.P., Berlin K., Miller J.R., Bergman N.H., Phillippy A.M. (2017). Canu: Scalable and accurate long-read assembly via adaptive k-mer weighting and repeat separation. Genome Res..

[30] Kolmogorov M., Yuan J., Lin Y., Pevzner P.A. (2019). Assembly of long, error-prone reads using repeat graphs. Nat. Biotechnol..

[31] Li H. (2018). Minimap2: Pairwise alignment for nucleotide sequences. Bioinformatics.

[32] Cock P.J., Antao T., Chang J.T., Chapman B.A., Cox C.J., Dalke A., Friedberg I., Hamelryck T., Kauff F., Wilczynski B. (2009). Biopython: Freely available Python tools for computational molecular biology and bioinformatics. Bioinformatics.

[33] Wong B. (2011). Points of view: Color blindness. Nat. Methods.

